# Can predictive factors determine the time to treatment initiation for oral and oropharyngeal cancer? A classification and regression tree analysis

**DOI:** 10.1371/journal.pone.0302370

**Published:** 2024-04-17

**Authors:** Débora Rosana Alves Braga Silva Montagnoli, Vitória Ferreira Leite, Yasmim Silva Godoy, Vitória Marçolla Lafetá, Edmilson Antônio Pereira Junior, Akhilanand Chaurasia, Maria Cássia Ferreira Aguiar, Mauro Henrique Nogueira Guimarães Abreu, Renata Castro Martins

**Affiliations:** 1 Graduate Program in Dentistry, School of Dentistry, Universidade Federal de Minas Gerais, Belo Horizonte, Brazil; 2 School of Dentistry, Universidade Federal de Minas Gerais, Belo Horizonte, Brazil; 3 Technical High School, Universidade Federal de Minas Gerais, Belo Horizonte, Minas Gerais, Brazil; 4 School of Education, Universidade Federal de Minas Gerais, Belo Horizonte, Brazil; 5 Department of Oral Medicine and Radiology, King George´s Medical University, Lucknow, Uttar Pradesh, India; 6 Department of Clinic, Dental Pathology and Surgery, School of Dentistry, Universidade Federal de Minas Gerais, Belo Horizonte, Brazil; 7 Department of Community and Preventive Dentistry, School of Dentistry, Universidade Federal de Minas Gerais, Belo Horizonte, Brazil; Universidade Federal do Rio Grande do Norte, BRAZIL

## Abstract

This ecological study aimed to identify the factors with the greatest power to discriminate the proportion of oral and oropharyngeal cancer (OOC) records with time to treatment initiation (TTI) within 30 days of diagnosis in Brazilian municipalities. A descriptive analysis was performed on the variables grouped into five dimensions related to patient characteristics, access to health services, support for cancer diagnosis, human resources, and socioeconomic characteristics of 3,218 Brazilian municipalities that registered at least one case of OOC in 2019. The Classification and Regression Trees (CART) technique was adopted to identify the explanatory variables with greater discriminatory power for the TTI response variable. There was a higher median percentage of records in the age group of 60 years or older. The median percentage of records with stage III and IV of the disease was 46.97%, and of records with chemotherapy, radiation, or both as the first treatment was 50%. The median percentage of people with private dental and health insurance was low. Up to 75% had no cancer diagnostic support services, and up to 50% of the municipalities had no specialist dentists. Most municipalities (49.4%) started treatment after more than 30 days. In the CART analysis, treatment with chemotherapy, radiotherapy, or both explained the highest TTI in all municipalities, and it was the most relevant for predicting TTI. The final model also included anatomical sites in the oral cavity and oropharynx and the number of computed tomography services per 100,000. There is a need to expand the availability of oncology services and human resources specialized in diagnosing and treating OOC in Brazilian municipalities for a timely TTI of OOC.

## Introduction

Oral and oropharyngeal cancers (OOC) are the sixth most common neoplasm in the world [1]. Brazil stands out for the high incidence of these cancer types. More than 15,000 new cases per year are estimated in the 2023–2025 period, ranking it eighth among the most frequent cancer types [2]. However, cases are still diagnosed at advanced stages, and delayed treatment is a reality in some populations, even with improved screening techniques and emerging therapies [3, 4].

Timely treatment of OOC involves access to health services influenced by individual and contextual determinants. Weller et al. [5] listed three contributors influencing the time intervals of pathways to begin cancer treatment: patient, disease, and health system factors.

The delayed onset of the treatment attributed to patients with OOC has been associated with sociodemographic aspects such as female gender and older age (over 60 years) [6, 7], low-level education, and low income [8]. Moreover, lack of knowledge about the disease, undefined interpretation of symptoms, cultural beliefs (among them, downplaying oral problems), and self-treatment also modulate the decision time to seek professional help [8].

The location and stage of the tumor and the type of treatment necessary influence the delay in beginning the treatment because of the disease [5]. OOC can have a slow and indolent evolution but with tumor and proliferative aggressiveness [9]. Thus, tumors located in more posterior regions of the mouth, such as the oropharynx, hard palate, and retromolar trigone, can be diagnosed at more advanced stages of the disease (III and IV) [6, 7, 10], which leads to the need for multimodal therapies (chemotherapy and radiotherapy) and lead to longer times for the start of OOC treatment [7, 10].

Concerning the health system, the adult population’s difficult access to dental care and the lack of coordinated actions at the different care levels (primary, secondary, and tertiary) [11, 12] can influence the late onset of treatment. The coordination of cancer care involves everything from health policies, organizational aspects of services, appropriate infrastructure, and prepared human resources to socioeconomic issues in the health system context [5].

Most of the Brazilian population depends on the public health system (Sistema Único de Saúde–SUS in Portuguese) [13]. The National Oral Health Policy has allowed the implementation of strategies for the early diagnosis of OOC. However, Brazil is plagued by marked social inequality, and some regions have incomplete access to oral health services [14].

Studies have already reported the influence of unequal population coverage of primary and secondary health services in Brazilian cities on elevated number of hospitalizations and deaths due to OOC, especially when public oral health services are absent [15, 16]. Therefore, the SUS-dependent population may have a time interval between diagnosis and treatment almost twice as long as private service patients [17].

The limited number of public facilities specializing in oncology, both diagnostic support and chemotherapy and radiotherapy equipment, is also a critical factor that has affected the increase in time to start cancer treatment in Brazil [18, 19]. Similarly, the need for qualified human resources for definitive early diagnosis in some municipalities is a challenge to overcome [20, 21]. Contacting an appropriate specialized health provider once the lesion was detected reduced diagnosis and treatment delay in cancer [22].

Furthermore, the unfavorable socioeconomic context of the Brazilian municipalities, such as the low human development Index (HDI), high inequality index, lower sanitation levels, and low education rates, were also associated with worse OOC outcomes [15, 23]. These local factors need to be better evaluated during treatment initiation.

The Brazilian law provides the right of patients with proven malignant neoplasm to undergo their first treatment within 60 days of diagnosis [24]. However, this Federal law is for all types of cancer and is not focused on oral cancer. The literature shows strong evidence that for OOC starting treatment after more than 30 days led to a worse prognosis and lower survival or a higher risk of death [7, 10, 25–27], stating that the processes involved in the care of these patients require particular attention. In patients with head and neck cancer, including oral cancer, waiting more than 60 days to start treatment affects survival by 26% compared to waiting less than 30 days [27].

Given this, the present study aimed to identify factors with the most significant power to discriminate the proportion of OOC records with time treatment initiation within 30 days after diagnosis in Brazilian municipalities. The alternative hypothesis was that individual and contextual factors are associated with municipalities’ records of longer treatment initiation times.

## Methods

**Fig 1:**
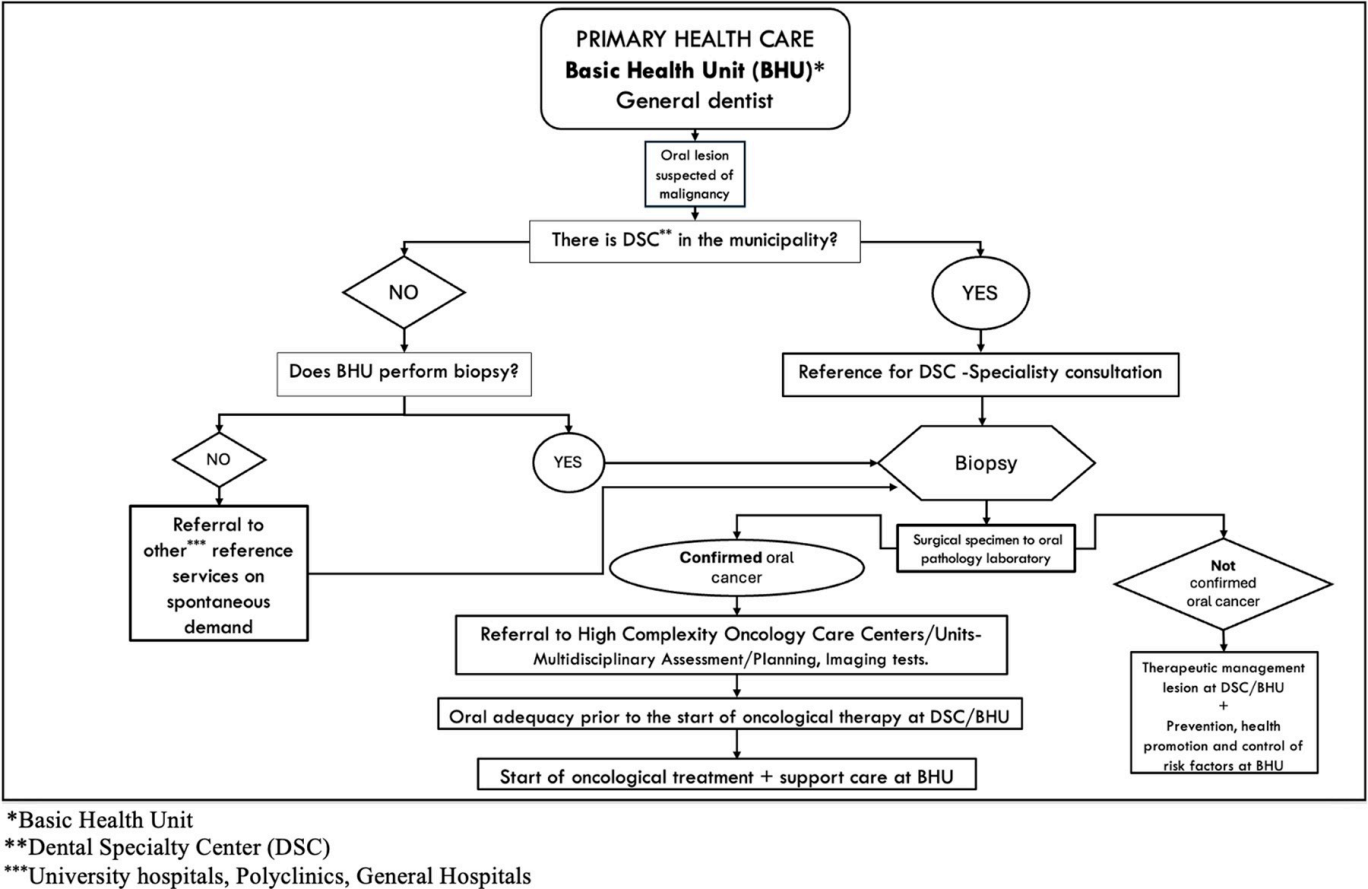
Flows of diagnosis and treatment of OOC in the Brazilian public health system.

This ecological study evaluated 3,218 Brazilian municipalities that registered at least one case of oral and oropharynx cancer in adults (20 years and over) in 2019. The municipal level organizes, executes, and manages the services and actions that occur in Primary Health Care (PHC) [28]. The PHC is the population’s main gateway to the Brazilian public health system and is responsible for coordinating flows between the many points of the Health Care Network (HCN) to ensure the integrality of care [28]. PHC, through the Basic Health Units (BHU), works on health promotion, prevention and control of risk factors, screening, and initial diagnosis of OOC [29]. When potentially malignant lesions are identified at PHC, patients are referred to secondary (Dental Specialties Centers—DSC) or tertiary levels of care for diagnostic confirmation through biopsy [12, 20, 29]. After confirming the diagnosis of malignancy, patients are referred to highly complex Oncology Centers or Units to undergo oncological surgery, radiotherapy, and/or chemotherapy. BHU also works on cancer pre-treatment adequacy procedures, as well as supporting patients during and after cancer treatment. In cases where the lesion is benign, the patient returns to the BHU for therapeutic management of the lesion, prevention, and control of risk factors [29]. Fig 1 shows the flows between the various points of the Brazilian HCN considering the path to diagnosis and treatment of OOC.

All the information is in the public domain. Therefore, the Human Research Ethics Committee did not need to approve this study. Secondary data on OOC registrations were obtained from the Oncology Treatment Monitoring Panel (Panel-Oncology) of the Informatics Department of the Unified Health System (DATASUS) (http://tabnet.datasus.gov.br/cgi/dhdat.exe?PAINEL_ONCO/PAINEL_ONCOLOGIABR.def) on February 2, 2023. This platform collects information from public health services (public or private health institutions affiliated with the SUS) that provide cancer care in Brazil. It is a tool for public health managers and health planners to monitor the time from diagnosis to first treatment, using data from the information systems consolidated in the SUS. Although it does not refer to the incidence of cancer in the country, it covers a high degree of heterogeneity in the population studied. The data are grouped by place of registration (municipalities, states, and macro-regions), and it is not possible to identify patients directly.

This study is based on the theoretical model of Weller et al. [5], in which targets are identified to facilitate the evaluation of the reasons interfering with early diagnosis and, consequently, improve the prognosis of cancer cases. To this end, three factors are considered–patient, health system, and disease–and modulate the decision-making processes that permeate the time intervals until treatment.

Time to treatment initiation (TTI) was the dependent variable and was defined as the proportion of municipal OOC records with treatment started within 30 days of confirmed diagnosis/total in 2019. Starting treatment after more than 30 days led to a worse prognosis and lower survival in OOC cases [7, 10, 25, 26, 27].

According to the Kolmogorov-Smirnov normality test, this variable showed a non-normal distribution (p >0.05). Thus, TTI was categorized into three groups: Delay: municipalities where all OCC treatments started after 30 days; 0<Delay<1: municipalities with OOC treatment records within 30 days and over 30 days; and No delay: municipalities where all OOC treatments started within 30 days.

The independent variables and their definitions, data source, and access are shown in Table 1. The explanatory variables were divided into five dimensions: 1. Patient characteristics (PC); 2. Access to health services (AS); 3. Cancer diagnosis support (CS); 4. Human resources (HR); and 5. Municipal characteristics (MC).

**Table 1:** Explanatory variables and their definitions, data source, and access, Brazil, 2019.

VARIABLE	DEFINITION	DATA SOURCE	ACCESS (internet)
**Patient Characteristics (PC)**			
PC1 –Percentage of OOC records aged 45 to 59	Total municipal OOC records (C00-C10) aged 45–59 by total municipal OOC records in 2019.	Panel-Oncology DATASUS	http://tabnet.datasus.gov.br/cgi/dhdat.exe?PAINEL_ONCO/PAINEL_ONCOLOGIABR.def
PC2 –Percentage of OOC records aged 60 or over	Total municipal OOC records (C00-C10) aged 60 or over by total municipal OOC records in 2019.	Panel-Oncology DATASUS	http://tabnet.datasus.gov.br/cgi/dhdat.exe?PAINEL_ONCO/PAINEL_ONCOLOGIABR.def
PC3 –Percentage of female OOC records	Total municipal female OOC records (C00-C10) by total municipal OOC records in 2019.	Panel-Oncology DATASUS	http://tabnet.datasus.gov.br/cgi/dhdat.exe?PAINEL_ONCO/PAINEL_ONCOLOGIABR.def
PC4 –Percentage of lip cancer	Total municipal lip cancer records (C00) by total municipal OOC records in 2019.	Panel-Oncology DATASUS	http://tabnet.datasus.gov.br/cgi/dhdat.exe?PAINEL_ONCO/PAINEL_ONCOLOGIABR.def
PC5 –Percentage of oral cavity cancer	Total municipal oral cavity cancer records (C02-C04, C06) by total municipal OOC records in 2019.	Panel-Oncology DATASUS	http://tabnet.datasus.gov.br/cgi/dhdat.exe?PAINEL_ONCO/PAINEL_ONCOLOGIABR.def
PC6 –Percentage of salivary gland cancer	Total municipal salivary gland cancer records (C07, C08) by total municipal OOC records in 2019.	Panel-Oncology DATASUS	http://tabnet.datasus.gov.br/cgi/dhdat.exe?PAINEL_ONCO/PAINEL_ONCOLOGIABR.def
PC7 –Percentage of oropharyngeal cancer	Total municipal oropharyngeal cancer records (C01, C05, C09, C10) by total municipal OOC records in 2019.	Panel-Oncology DATASUS	http://tabnet.datasus.gov.br/cgi/dhdat.exe?PAINEL_ONCO/PAINEL_ONCOLOGIABR.def
PC8 –Percentage of OOC staging III and IV	Total municipal OOC records (C00-C10) with stages III and IV by total municipal OOC records in 2019.	Panel-Oncology DATASUS	http://tabnet.datasus.gov.br/cgi/dhdat.exe?PAINEL_ONCO/PAINEL_ONCOLOGIABR.def
PC9 –Percentage of OOC treated with chemotherapy, radiotherapy, or both	Total municipal OOC records (C00-C10) treated with chemotherapy, radiotherapy, or both by total municipal OOC records in 2019.	Panel-Oncology DATASUS	http://tabnet.datasus.gov.br/cgi/dhdat.exe?PAINEL_ONCO/PAINEL_ONCOLOGIABR.def
**Access to Health Services (AS)**			
AS1 –Presence of Dental Specialties Centers (DSC)	DSC is active through December 2019	National Health Facilities Census DATASUS	https://cnes.datasus.gov.br/pages/estabelecimentos/consulta.jsp
AS2 –Coverage of Family Health Teams with oral health teams	Average coverage for 2019.	e-Manager Basic Care	https://egestorab.saude.gov.br/paginas/acessoPublico/relatorios/relHistoricoCoberturaSB.xhtml
AS3- Coverage of Primary Care Oral Health Teams	Average coverage for 2019.	e-Manager Basic Care	https://egestorab.saude.gov.br/paginas/acessoPublico/relatorios/relHistoricoCoberturaSB.xhtml
AS4- Percentage of population with private dental insurance	Total population of the municipality with a dental plan in 2019 by the municipality’s population estimate for 2019.	National Supplementary Health Agency DATASUS	https://www.ans.gov.br/anstabnet/cgi-bin/dh?dados/tabnet_tx.def
AS5- Percentage of population with health insurance—medical assistance	Total population of the municipality with health insurance in 2019 by the municipality’s population estimate for 2019.	National Supplementary Health Agency DATASUS	https://www.ans.gov.br/anstabnet/cgi-bin/dh?dados/tabnet_tx.def
AS6- Percentage of SUS beds in the municipality per 100,000 inhabitants	Total number of surgical beds (oral and maxillofacial surgery, general surgery, and oncology) and clinical beds (general medicine and oncology) available in public and private hospitals affiliated with the SUS in 2019, based on the municipality’s 2019 population estimate.	National Health Facilities Census DATASUS	https://cnes2.datasus.gov.br/Mod_Ind_Tipo_Leito.asp?VEstado=11&VMun=110032&VComp=201909
**Cancer Diagnosis Support (CS)**			
CS1 –Number of clinical oncology services per 100,000 inhabitants	Total surgical oncology services (outpatient and hospital) available in the municipality based on the municipality’s population estimate for 2019 x 100,000 inhabitants.	National Health Facilities Census DATASUS	https://cnes2.daasus.gov.br/Mod_Ind_Especialidades.asp?VEstado=11&VMun=110001&VComp=00&VTerc=00&VServico=132&VClassificacao=004&VAmbu=&VAmbuSUS=1&VHosp=&VHospSUS=1
CS2 –Number of radiotherapy services per 100,000 inhabitants	Total radiotherapy services (outpatient and hospital) available in the municipality based on the municipality’s population estimate for 2019 x 100,000 inhabitants.	National Health Facilities Census DATASUS	https://cnes2.daasus.gov.br/Mod_Ind_Especialidades.asp?VEstado=11&VMun=110001&VComp=00&VTerc=00&VServico=132&VClassificacao=004&VAmbu=&VAmbuSUS=1&VHosp=&VHospSUS=1
CS3 –Number of anatomopathological diagnostic services per 100,000 inhabitants	Total number of general hospitals, clinics and laboratories specializing in pathological diagnosis available in the municipality, with SUS service, based on the municipality’s population estimate for 2019 x 100,000 inhabitants.	National Health Facilities Census DATASUS	https://cnes2.datasus.gov.br/Mod_Ind_Especialidades.asp?VEstado=11&VMun=110001&VComp=00&VTerc=00&VServico=132&VClassificacao=004&VAmbu=&VAmbuSUS=1&VHosp=&VHospSUS=1
CS4 –Number of MRI diagnostic services per 100,000 inhabitants	Total number of hospitals, centers and clinics specialized in magnetic resonance imaging (MRI) diagnosis available in the city, with SUS service, based on the municipality’s population estimate for 2019 x 100,000 inhabitants.	National Health Facilities Census DATASUS	https://cnes2.datasus.gov.br/Mod_Ind_Especialidades.asp?VEstado=11&VMun=110001&VComp=00&VTerc=00&VServico=132&VClassificacao=004&VAmbu=&VAmbuSUS=1&VHosp=&VHospSUS=1
CS5 –Number of CT imaging diagnostic services per 100,000 inhabitants	Total hospitals, centers, and clinics specializing in computed tomography (CT) diagnosis available in the city, with SUS service based on the municipality’s population estimate for 2019 x 100,000 inhabitants.	National Health Facilities Census DATASUS	https://cnes2.datasus.gov.br/Mod_Ind_Especialidades.asp?VEstado=11&VMun=110001&VComp=00&VTerc=00&VServico=132&VClassificacao=004&VAmbu=&VAmbuSUS=1&VHosp=&VHospSUS=1
**Human Resources (HR)**			
HR1 –Number of oral medicine specialists and oral pathologists per 100 thousand inhabitants	Total number of oral medicine specialists and oral pathologists in the municipality according to the municipality’s population estimate for 2019 x 100,000 inhabitants.	National Health Facilities Census DATASUS	https://cnes2.datasus.gov.br/Mod_Ind_Tipo_Leito.asp?VEstado=11&VMun=110032&VComp=201909
HR2 –Number of maxillofacial surgeons per 100 thousand inhabitants	Total number of maxillofacial surgeons in the municipality based on the municipality’s population estimate for 2019 x 100,000 inhabitants.	National Health Facilities Census DATASUS	https://cnes2.datasus.gov.br/Mod_Ind_Tipo_Leito.asp?VEstado=11&VMun=110032&VComp=201909
HR3 –Number of specialist dentists per 100,000 inhabitants	Total number of oral medicine specialists, oral pathologists and maxillofacial surgeons in the city based on the municipality’s population estimate for 2019 x 100,000 inhabitants.	National Health Facilities Census DATASUS	https://cnes2.datasus.gov.br/Mod_Ind_Tipo_Leito.asp?VEstado=11&VMun=110032&VComp=201909
**Municipal Characteristics (MC)**			
MC1 –Municipal Human Development Index (M-HDI)	M-HDI Census 2010.	Atlas Brazil	http://www.atlasbrasil.org.br/ranking
MC2 –Gini index of household income per capita	Gini index of household income per capita Census 2010.	IBGE	http://tabnet.datasus.gov.br/cgi/ibge/censo/cnv/ginibr.def
MC3 –Municipal Gross Domestic Product (GDP)	GDP- 2019.	IBGE	https://www.ibge.gov.br/estatisticas/economicas/contas-nacionais/9088-produto-interno-bruto-dos-municipios.html?t=downloads&c=1100015
MC4 –Illiteracy rate among the population aged 15 and over	Municipality Census 2010.	Atlas Brazil	http://www.atlasbrasil.org.br/consulta/planilha
MC5-Unemployment rate for the population aged 18 and over	Municipality Census 2010.	Atlas Brazil	http://www.atlasbrasil.org.br/consulta/planilha
MC6 –Percentage of people in urban households with garbage collection	Municipality Census 2010.	Atlas Brazil	http://www.atlasbrasil.org.br/consulta/planilha
MC7 –Percentage of people in households with inadequate water supply and sewage	Municipality Census 2010.	Atlas Brazil	http://www.atlasbrasil.org.br/consulta/planilha

Variables were collected from the Panel-Oncology according to aspects most associated with delay in OOC treatment reported in the literature to build the Patient Characteristics (PC) dimension, such as female sex, age groups most affected by the disease (45–59 and above 60 years), more advanced stages (III and IV), treatment with chemotherapy, radiotherapy, or both [6, 7, 10]. The proportions of records for each variable by the total number of records for each municipality in 2019 were calculated. Data were collected from the place of residence of registered cases.

Oral neoplasm records are identified using the C00-C10 classification of the 10^th^ revision of the International Classification of Diseases (ICD-10) in the Panel-Oncology. When analyzing oral cancer, some authors advocate excluding lip and salivary gland neoplasms from this category because they have different etiologic, histologic, and epidemiologic behavior [30, 31], which affects the time to start treatment. Because of this, the proportion of OOC records was evaluated by groups of anatomical sites separately: PC4 (lip cancer: C00), PC5 (oral cavity: C02-C04 and C06), PC6 (salivary glands: C07 and C08), and PC7 (oropharynx: C05, C09, and C10) (Table 1).

As for the dimension of CS, only public or private services affiliated with the SUS were considered. For the CS1 variable, all clinical oncology services (outpatient and inpatient) available in the municipalities were considered. Pediatric hospitals were excluded because of the rare oral malignant neoplasms in children [32]. For the CS3 variable, general hospitals, clinics, and specialized pathology diagnostic laboratories available in the municipality were considered. Essential health or primary care and mixed units were excluded, as these generally perform cytopathology tests related to cervical cancer control and prevention. Hospitals limited to a single medical specialty (e.g., nephrology, ophthalmology, gynecology, neurology, and urology) were also excluded (Table 1).

The socioeconomic characteristics of the municipalities were obtained from the Brazilian Institute of Geography and Statistics (IBGE) census 2010. Municipal population estimates for 2019 were taken from the IBGE to calculate percentages per 100,000 inhabitants to obtain the distribution ratios of health services provided by municipalities.

A descriptive analysis of the explanatory variables was carried out to evaluate the median, P25%, P75% and minimum and maximum values. Next, the “Classification and Regression Trees” (CART) technique was performed to identify the explanatory variables, distributed across five dimensions, which presented greater discrimination power for the TTI response variable. This technique consists of a decision tree in which data is divided into smaller groups based on specific rules, creating a hierarchy of decisions in which previous divisions affect subsequent ones. The logic of this technique is based on building the tree through the successive subdivision of groups into subgroups [33]. These consecutive dataset splits are performed using the Chi-square Automatic Interaction Detection-CHAID method. The procedure selects the independent variable with the most significant interaction with the dependent variable at each subdivision. Thus, it is possible to identify homogeneous subgroups, reducing the effects of multicollinearity, outliers, and missing data.

It was necessary to establish some criteria in advance when modeling the data: 1) each node (a designation given to each subset resulting from the application of a division rule) should contain a minimum of 500 observations; 2) terminal nodes should include at least 200 observations; 3) exclusion of subdivisions with significance probability values (p-value) greater than 0.05. The algorithm automatically established cutoff points to segment the analyzed groups. This analysis was also carried out for the five Brazilian regions. All analyses were performed using the Statistical Package for Social Sciences (SPSS), version 22.0 (IBM SPSS Statistics for Windows, Armonk, NY).

## Results

In 2019, 57.79% of all municipalities in Brazil (n = 5,568) recorded at least one case of OOC. Table 2 shows the descriptive analysis of the explanatory variables of the five dimensions.

**Table 2:** Descriptive analysis of variables related to patient characteristics (PC), access to health services (AS), cancer diagnosis support (CS), human resources (HR), and municipal characteristics (MC).

Variable	Median	P25%	P75%	Min.	Max.
**Patient characteristics (PC)**					
PC1	33.33	0.00	60.00	0.00	100.00
PC2	50.00	0.00	100.00	0.00	100.00
PC3	14.29	0.00	50.00	0.00	100.00
PC4	0.00	0.00	0.00	0.00	100.00
PC5	33.33	0.00	50.00	0.00	100.00
PC6	0.00	0.00	12.50	0.00	100.00
PC7	40.00	0.00	66.67	0.00	100.00
PC8	46.97	0.00	75.00	0.00	100.00
PC9	50.00	20.00	100.00	0.00	100.00
**Access to health services (AS)**					
AS2	79.14	36.88	100.00	0.00	100.00
AS3	91.66	55.23	100.00	0.00	100.00
AS4	2.49	0.99	6.98	0.00	2,460.33
AS5	6.43	1.89	16.15	0.00	3,775.51
AS6	5.72	0.00	10.99	0.00	245,86
**Cancer diagnosis support (CS)**					
CS1	0.00	0.00	0.00	0.00	16.72
CS2	0.00	0.00	0.00	0.00	16.72
CS3	0.00	0.00	0.00	0.00	94.56
CS4	0.00	0.00	0.00	0.00	63.45
CS5	0.00	0.00	0.00	0.00	63.45
**Human resources (HR)**					
HR1	0.00	0.00	0.00	0.00	200.18
HR2	0.00	0.00	1.72	0.00	709.22
HR3	0.00	0.00	2.05	0.00	709.22
**Municipal characteristics (MC)**					
MC1	0.69	0.62	0.73	0.48	0.86
MC2	0.50	0.46	0.54	0.28	0.78
MC3	20,605.71	11,785.13	32,327.55	4,595	484,883.50
MC4	11.07	6.84	22.20	0.95	44.40
MC5	5.88	3.92	8.10	0.09	25.26
MC6	98.42	95.34	99.54	16.25	100.00
MC7	2.35	0.46	10.67	0.00	72.22

### Patient characteristics (PC)

There was a higher median percentage of records in the age group of 60 years or more (PC2) (50.00;0–100); oropharyngeal cancer (PC7) (40; 0–100) and oral cavity cancer (PC5) (33.33;0–100). The median percentage of records in stages III and IV (PC8) was 46.97% (0–100), and of records that had chemotherapy, radiotherapy, or both (PC9) as their first treatment was 50% (0–100).

### Access to health services (AS)

Approximately 73.4% of municipalities did not have active DSC in 2019 (Results not shown).

The median coverage of Family Health Teams was 79.14% (AS2) and 91.66% for Oral Health in Primary Care (AS3), ranging from zero to 100%. However, the median percentage of people with private dental insurance in municipalities (AS4) (2.49; 0 to 2,460.331) and health insurance (AS5) (6,43; 0 to 3,775,508) was low. The median number of SUS beds (AS6) was 5.72, ranging from zero to 245 beds per 100,000 inhabitants.

### Cancer diagnosis support (CS)

The municipalities had a maximum of 14.98 surgical oncology services; 16.72 clinical oncology (CS1) and radiotherapy (CS2) services; 94.56 anatomopathological diagnoses (CS3) services; and 63.45 MRI (CS4) and CT (CS5) imaging diagnosis services per 100,000 inhabitants.

### Human resources (HR)

Up to 50% of the municipalities needed a specialist dentist. The maximum value found was 709.22 specialist dentists (HR3) per 100,000 inhabitants.

### Municipal characteristics (MC)

In general, the municipalities had different socioeconomic profiles. The median HDI-M (MC1) revealed medium development, ranging from low to high (0.69; 0.48–0.86). Similarly, the municipalities had a median illiteracy rate for the population aged 15 or over (MC4) of 11.07%, ranging from 0.95% to 44.40%; a median unemployment rate for people aged 18 or over (MC5) of 5.88% (0.09–25.26) and a median percentage of people in households with inadequate water supply and sewage (MC7) of 2.35%, ranging from 0 to 72.22%.

**Fig 2:**
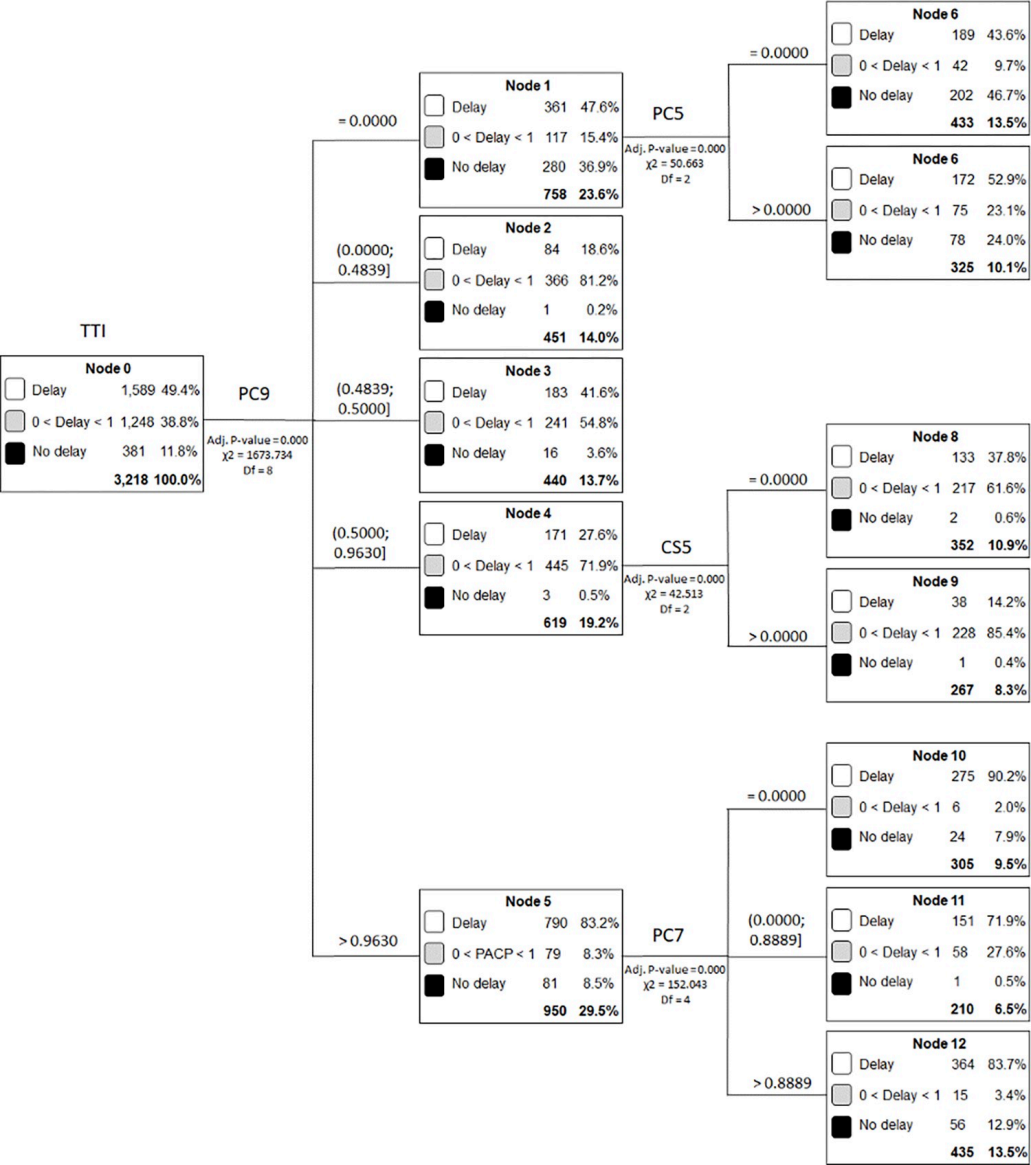
CART analysis for time to treatment initiation of Brazilian municipalities.

CART models for Brazilian municipalities are shown in Fig 2.

Most municipalities (49.4%) started treatment after more than 30 days (Delay group), and 11.8% of municipalities started treatment within 30 days (No delay group).

Treatment with chemotherapy, radiotherapy, or both (PC9) explained the highest TTI in all municipalities; it was the most relevant method for predicting the TTI. When most patients (PC9>0.963) were treated by chemotherapy, radiotherapy, or both, a delay of more than 30 days was seen in 83.2% of patients.

In the decision tree (Fig 2), Node 6 was the only one that showed most municipalities in the "No delay" group of TTI (46.7%). This node shows that when there was no record of first treatment by chemotherapy, radiotherapy, or both (Node 1; PC9 = 0) and, at the same time, there was no record with anatomical location in the oral cavity (Node 6; PC5 = 0), most municipalities recorded the start of treatment within 30 days.

Anatomical locations in the oral cavity (PC5) and oropharynx (PC7), and the number of CT imaging services (CS5) available in the municipality per 100,000, were also included in the final model.

**Fig 3:**
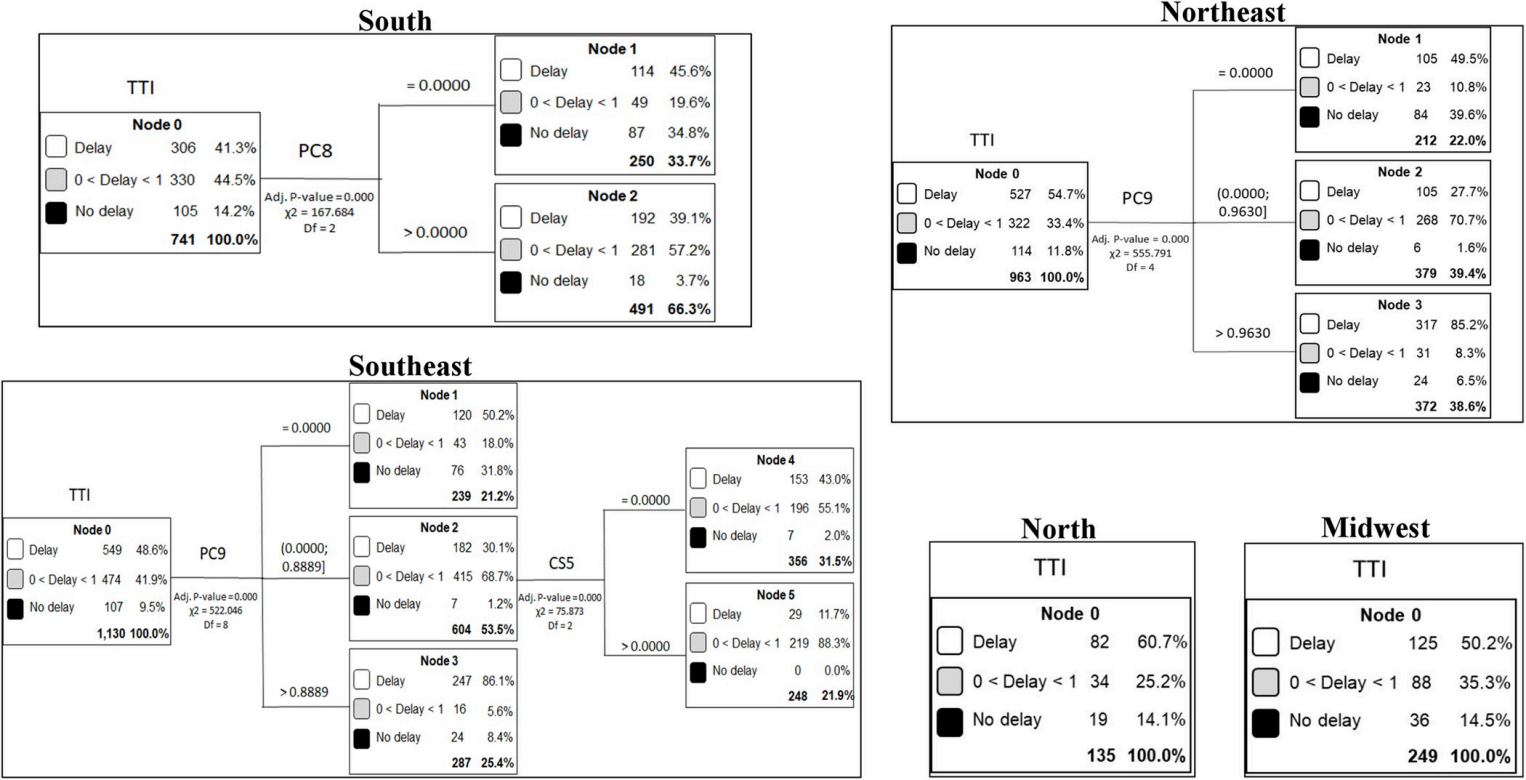
CART analysis for time to treatment initiation for Brazilian regions.

Fig 3 shows the CART analyzes for Brazilian regions.

South region had 741 municipalities with OOC records. Most municipalities of South region (41.3%) started treatment after more than 30 days (Delay group), and 14.2% of municipalities started treatment within 30 days (No delay group). In this region the staging III and IV (PC8>0.0001) explained the highest TTI in the most of municipalities (n = 491). When the patients (PC8>0.0001) presented staging III and IV a delay of more than 30 days was seen in 39.1% of patients.

In the Southeast, 1,130 municipalities reported cases of OOC. Most municipalities of South region (48.6%) started treatment after more than 30 days (Delay group), and 9.5% of municipalities started treatment within 30 days (No delay group). This region showed two levels of subdivision. The highest TTI was explained by treatment with chemotherapy, radiotherapy, or both (PC9). At Node 2, a subdivision was created where the number of CT imaging services (CS5) available in the municipality per 100,000 explained most municipalities in the 0<Delay<1 group (88.3%).

In the Northeast region, 963 municipalities reported at least one case of OOC. Of these, 54.7% were classified in the ’Delay’ group. The highest TTI in Northeast municipalities was explained by treatment with chemotherapy, radiotherapy, or both (PC9).

North (60.7%) and Midwest (50.2%) regions had the majority of municipalities in the "Delay" group. These regions were not subdivided as they did not meet the minimum methodological criteria established for the model once the terminal nodes should include at least 200 observations.

## Discussion

Most municipalities delayed the TTI. Chemotherapy or radiotherapy treatment, the number of CT imaging services per 100,000 inhabitants, and anatomical location of cancer classified as oral cavity and oropharynx were associated with the higher OOC TTI in the municipalities.

The higher percentage of records for the 60 or more age group in this study corroborates the literature on this type of cancer in older people [7, 8]. The anatomical classification comprising the oropharynx (base of tongue, palate, tonsil, and oropharynx) and oral cavity (unspecified parts of tongue and mouth, floor of mouth, and gums) were the most prevalent and explained a higher TTI among the municipalities in this study in the CART model. OCCs located on the floor of the mouth and of the tongue are the most frequent types of oral cavity cancer already reported [34]. However, oropharyngeal cancer showed the most significant increase in incidence in Brazil [35]. OOC in these sites, coupled with slow and indolent growth, makes it challenging to identify the lesion, which means that patients do not seek professional help in the early stages of the disease and delays the treatment pathway [8]. Also, some OOC types, especially no-HPV, tend to metastasize to the cervical lymph nodes early without noticeable primary lesions, symptoms, or changes in the mucous membranes [36]. Therefore, some professionals may underestimate the assessment of malignancy and incur delays in referral to the specialized service [8].

Given this context, most OOC cases are still diagnosed at stages III and IV in Brazil [37]. The staging III and IV was associated to increases in TTI in South of Brazil. These more advanced stages of the disease require more complex or multimodal therapies involving chemotherapy and radiotherapy [36]. Treatment by these therapeutic modalities (chemotherapy, radiotherapy, or both) explained the highest TTI for all the municipalities in this study, especially among municipalities in the Southeast and Northeast regions. When this type of treatment is considered, delays can be explained by more time for planning, primarily due to complications associated with tobacco and alcohol use, the need to manage adjacent comorbidities pre-treatment, a more significant number of complementary tests, oral adequacy (such as tooth extractions), referrals to other treatment support professionals and rigorous planning by multidisciplinary teams [38, 39].

Considering the situation of Brazilian public health, the delay in OOC explained by chemotherapy and radiotherapy treatment may be related to the low availability of cancer support services denoted in the municipalities of this study. In 2019, Ordinance N° 1.399 was defined as a parameter for enabling Brazilian oncology services, at least one facility per 1,000 new cancer cases per year (except for non-melanoma skin cancer) [40]. However, there is still a need for more machines, especially radiotherapy machines [19], and the geographical distribution seems inadequate for all regions [41]. Other studies on cervical cancer [42], breast cancer [18], and lung cancer [19] showed that place of residence affected TTI, confirming the unequal availability of public oncology services in Brazil. High-complexity services are concentrated in large urban centers, aligning with local economic development [41]. It is no wonder that more developed Brazilian regions have reduced the risk of death from OOC, while less developed regions have shown an increase in recent cohorts [43].

The regions with the highest number of municipalities with OOC records are the Southeast and Northeast, which are also the most populous regions in the country [18]. This suggests that the high demand from patients leads to longer waiting times for chemotherapy/radiotherapy treatment in Brazil [18, 41], even in regions such as the Southeast, which, compared to the other, has the greatest technological support for cancer treatment [14, 19, 41]. The Northeast region, however, has a high concentration of municipalities with low socioeconomic development and barriers to accessing health services are related to poor income distribution and social inequality [15, 43].

Therefore, due to the complexity of cancer treatment, it is crucial for municipalities to have well-established care flows to ensure timely OOC treatment in a high-demand patient [12, 16, 20, 42]. Lack of supply or coverage of services, professionals and infrastructure that make it difficult for patients with OOC to access this care network can lead to delays in treatment [12, 15–17]. In addition, the arrangement of chemotherapy and radiotherapy services should consider epidemiological and social factors, the number of qualified professionals, and the capacity of each unit. This is especially important for serving the socioeconomically disadvantaged population that is also the most affected by OOC [34].

In general, the centralization of chemotherapy and radiotherapy facilities in specific reference units subjects patients to long-distance travel, poor road conditions, or unsatisfactory means of transport, and, in the last resort, the imposition of relocating their place of residence, especially in a country as continental as Brazil [41]. The national average distance traveled by cancer patients for treatment varies between 105.8 and 117.1 miles (170.3 and 188.4 km, respectively), with regional disparities, where patients from the North and Midwest travel greater distances than patients in the South, Southeast, and Northeast [14], which can influence limit treatment options and hinder managing side effects [44, 45]. In other words, it creates barriers to accessing health services, thus delays treatment [10, 39], and leads to worse survival outcomes in OOC cases [45].

The unavailability of radiotherapy services is not exclusive to Brazil. An Italian study showed a higher TTI for patients who needed radiotherapy because the devices were only available in research/academic institutes, generating long waiting lines and patient selection [38]. Infrastructural equipment maintenance failures and lack of radiation oncologists are the main factors in delays in treatment in low and medium-development countries [46]. The difficult access to radiotherapy in low-resource or rural settings may have been associated with higher mortality in these regions [47].

A similar problem occurs in the distribution of CT imaging services, which also explains a higher TTI among municipalities, especially those in the 0<Delay<1 group. This suggests that the municipality favors lower TTI when it offers CT facilities. On the other hand, the unavailability of these facilities, which is the reality for most municipalities in this study, leads to long waiting lines for appointments and the need for patients to travel to undergo the tests and for returns and appointments. In cases where intensity-modulated radiotherapy (IMRT) is indicated, for example, there is a greater need for CT simulations for case planning and, therefore, greater ITT [48]. One study showed that in Brazil, the degree of use of CT scanners is low in relation to the quantity available in the public health sector, showing that there are public management and infrastructure problems that need to be overcome, ranging from a lack of qualified professionals to operate the equipment, devices that are out of service for long periods of time, to problems with the electricity supply in the unit or region, for example [49].

The National Policy for Cancer Prevention and Control advocates that cancer care be structured and organized into a line of care that encompasses all care levels in order to guarantee comprehensive care in HCN [50], which incorporates flows and connections for referring patients to different territories. Thus, primary care professionals are responsible for preventing and controlling risk factors and screening for potentially malignant lesions, which should be referred to specialized services to confirm the diagnosis [51]. Although this study’s primary care coverage rate is satisfactory, oral health teams hardly conduct OOC-related actions in the country [52, 53]. This is because dentists feel unable to identify a potentially malignant lesion and perform biopsies [54], mainly because these are rare lesions to see throughout one’s career [55]. However, primary care dentists must be trained to recognize the premalignant lesions early as a clinical routine to reduce delays in OOC diagnosis and treatment [54].

Secondary care facilities and specialized human resources need to be adequately available and distributed to meet the Brazilian OOC demand. In this study, more than 70% of municipalities did not have active DSCs in 2019. DSC is the leading public service for access to OOC diagnosis, where oral medicine is offered as a minimum and mandatory specialty [20, 56]. However, DSC support for primary care within the oral healthcare network still needs to be improved since only some carry out all the recommended actions [57]. From 2014 to 2018, there was a reduction in the number of DSCs that performed biopsies [12], meaning that there is a problem with the distribution of DSCs in the country, and only some of them participate in the OOC patient care network. This is very troubling, given that the shortage of DSCs has been linked to a higher risk of hospitalization and death from oral cancer [15]. Furthermore, although Brazil has the most significant number of dental professionals globally, less than 1% are oral medicine specialists [58]. There is also a marked leaving of these professionals from DSC, mainly due to the need for more resources, supplies, and work equipment, for example, to perform biopsies [20].

Studies have already reported that the greater availability of public oral health services has contributed to a lower frequency of stage IV OOC [16], and the expansion of primary dental care coverage and the number of dental specialty centers are associated with lower mortality rates from OOC in Brazil [51]. Therefore, acting on bottlenecks that influence diagnosis delays is one way to prevent cases from being identified at advanced stages, which can reduce ITT and improve patient survival [59].

Besides the need for more service structures and human resources for OOC care in Brazil, there are areas for improvement in referral and counter-referral flows for OOC prevention, diagnosis, and treatment [60]. The lack of secondary services means that patients with suspected malignant oral lesions are referred to other services through informal and direct contacts [12], such as academic units. However, many of these units are not linked to public health management, leading to gaps in the line of care and the treatment pathway. In addition, many DSCs have difficulties making hospital referrals for confirmed OOC cases [12], and given the high cost of treatment, patients may seek independent access to reference oncology services or face long waiting lists [14].

The coordination of OOC care is complex as it is a type of cancer that requires the involvement of different professionals, from clinical dentists, oral medicine specialists, oral and maxillofacial surgeons, head and neck surgeons, oncologists, among others, who need to be supported by effective government incentive programs. This will require improvements in care integration and the organization and distribution of medium and high-complexity services. Investing in physical infrastructure and human resources is necessary to achieve this. Financial incentives in the human resources of the DSC favor collaborative relationships for articulation between primary and secondary care, improving the quality of dental care [57]. Mortality from OOC has decreased in the more socioeconomically developed Brazilian regions, where there has been more significant government investment in outpatient procedures and hospitalization [61]. Therefore, there is an urgent need to increase public investment in oral health to ensure timely treatment of OOC. This overview will help health managers at different levels of government to plan effective interventions that prioritize the most vulnerable areas.

This study is unique in that it is the first, as far as we know, to use municipalities as the unit of analysis, showing a general overview of the factors that explain the TTI of OOC in Brazil and Brazilian regions. Data are robust as official information systems consolidate them.

At the same time, it has limitations inherent to its ecological design and the use of secondary data sources. The study considered data from population aggregates, and ecological fallacy can occur if its results are interpreted at individual levels. The National Registry of Health Establishments is one of the main repositories of information about the Brazilian health system. Registration and maintenance of registration data in the National Register of Health Establishments is mandatory for all health establishments to operate in the national territory [62]. Unfortunately, there may be inconsistencies or inaccuracies in the information provided by secondary data systems like this one about the real situation of health services. The system records may be completed incorrectly or after the deadline stipulated by the Ministry of Health, resulting in possible underreporting of cases or incomplete/inconsistent information. However, the databases were checked by two senior researchers for possible inconsistencies and nothing was found. Previous articles have confirmed the reliability of this database [41, 63, 64].

The study of the factors involving the ITT OOC deserves to be more detailed since the delay in treatment is linked to the delay in the system. Brazilian public health management is decentralized and depends on political agreements between managers in each region, which gives it some particularities in the availability of health services that need to be studied more closely to understand the events that lead to an increase in TTI. Moreover, this study showed a pre-pandemic reality in which health services were theoretically functioning correctly. The pandemic is known to have affected delays in cancer diagnosis and treatment in Brazil [65]. Studies evaluating post-pandemic services are also critical.

## Conclusion

Most municipalities delayed the TTI. Type of treatment chemotherapy, radiotherapy, tumor location (oral cavity and oropharynx), and factors to cancer diagnostic support (number of services with diagnostic imaging per 100,000 inhabitants) explained the higher TTI. The explanatory variables for the increase in TTI in the municipalities, even if indirectly, are connected to system factors. Therefore, managing the timely treatment of OOC requires expanding actions and services for diagnosis and treatment in Brazilian municipalities.

## Supporting information

S1 File Dataset. (XLSX)
